# Breastfeeding advice for reality: Women's perspectives on primary care support in South Africa

**DOI:** 10.1111/mcn.12877

**Published:** 2019-08-12

**Authors:** Tanya Doherty, Christiane Horwood, Lyn Haskins, Vuyolwethu Magasana, Ameena Goga, Ute Feucht, David Sanders, Thorkild Tylleskar, Shuaib Kauchali, Muhammad Ali Dhansay, Nigel Rollins, Max Kroon, Ingunn M. S. Engebretsen

**Affiliations:** ^1^ Health Systems Research Unit South African Medical Research Council Cape Town South Africa; ^2^ School of Public Health University of the Western Cape Cape Town South Africa; ^3^ Centre for Rural Health University of KwaZulu‐Natal Durban South Africa; ^4^ Department of Paediatrics University of Pretoria Pretoria South Africa; ^5^ Research Centre for Maternal, Fetal, Newborn and Child Health Care Strategies University of Pretoria Pretoria South Africa; ^6^ Maternal and Infant Health Care Strategies Research Unit South African Medical Research Council Pretoria South Africa; ^7^ Department of Paediatrics and Child Health University of Cape Town Cape Town South Africa; ^8^ Centre for International Health University of Bergen Bergen Norway; ^9^ Department of Health Pretoria South Africa; ^10^ Burden of Disease Research Unit South African Medical Research Council Cape Town South Africa; ^11^ Division of Human Nutrition and Department of Paediatrics and Child Health, Faculty of Medicine and Health Sciences Stellenbosch University Stellenbosch South Africa; ^12^ Department of Maternal, Newborn, Child and Adolescent Health World Health Organization Geneva Switzerland; ^13^ Department of Neonatology, Faculty of Health Sciences University of Cape Town and Mowbray Maternity Hospital Cape Town South Africa

**Keywords:** breastfeeding, health worker, infant feeding, primary health care, qualitative, women

## Abstract

Breastfeeding education and support are critical health worker skills. Confusion surrounding infant feeding advice linked to the HIV epidemic has reduced the confidence of health workers to support breastfeeding. High antiretroviral therapy coverage of breastfeeding women living with HIV, and an Infant Feeding policy supportive of breastfeeding, now provides an opportunity to improve breastfeeding practices. Challenges remain in restoring health worker confidence to support breastfeeding. This qualitative study presents findings from focus group discussions with mothers of young infants, exploring their experiences of health worker breastfeeding counselling and support. Analysis followed the thematic framework approach. Six researchers reviewed the transcripts, coded them independently, then jointly reviewed the codes, and agreed on a working analytical framework. Although mothers received antenatal breastfeeding messages, these appeared to focus rigidly on the importance of exclusivity. Mothers described receiving some practical support with initiation of breastfeeding after delivery, but support and advice for post‐natal breastfeeding challenges were often incorrect or absent. The support also ignored the context in which women make infant feeding decisions, including returning to work and pressures from family members. Despite improved breastfeeding policies, restoring confidence in health workers to support breastfeeding remains a challenge. The post‐natal period, when mothers experience breastfeeding difficulties, is particularly critical, and our findings reinforce the importance of continuity of care between communities and health facilities. This research has implications for how health workers are trained to support breastfeeding. Greater attention is needed on developing skills and confidence in identifying, assessing, and supporting women experiencing breastfeeding challenges.

**Key messages**

- Breastfeeding education and support are critical health worker skills; however, confusion surrounding infant feeding advice linked to the HIV epidemic has reduced the confidence of health workers to support breastfeeding.
- Whilst women described antenatal group information, postnatal breastfeeding support appeared to ignore the context in which women make decisions, including returning to work and pressures from family members. Health worker counselling appeared not to anticipate and prepare mothers for these challenges.
- Continuity of care between communities and health facilities is critical. Greater attention should be paid to developing health worker confidence in assessing and supporting women experiencing common breastfeeding challenges.

## 1 INTRODUCTION

There are significant health, societal, and economic benefits from high coverage of exclusive breastfeeding (EBF). However, in low‐ and middle‐income countries, only 37% of children younger than 6 months of age are exclusively breastfed (Victora et al., 2016). The Global Nutrition Target (World Health Assembly) is to increase the EBF rate in the first 6 months to at least 50% by 2025 (WHO/UNICEF, 2014). Providing information and support for breastfeeding is a critical skill required of health workers in all settings, including primary health care (PHC). Health workers are key providers of infant feeding messages, and mothers often make decisions based primarily on feeding advice from health workers, despite pressures from family and the wider community (Fjeld et al., 2008; Horwood, Jama, Haskins, Coutsoudis, & Spies, 2019). Many studies have shown that counselling from health workers throughout the antenatal and breastfeeding period can effectively improve feeding practices (Rollins et al., 2016). In low‐ and middle‐income countries contexts, this often takes the form of individual or group information during antenatal care and intermittent assistance with initiation and attachment immediately post‐delivery, if health worker skills and time permit. The reality is, however, that women are commonly discharged within a few hours of delivery, so there is little time available for immediate post‐natal support.

The emergence of the HIV epidemic and subsequent evidence of HIV transmission through breastmilk led to confusion among health workers and inconsistency in the information given to women due to multiple interpretations of guidelines. Since 2016, the international guidance has recommended EBF for 6 months and continued breastfeeding until 2 years or longer in the context of antiretroviral therapy, thus aligning the infant feeding recommendations for all women irrespective of their HIV status (World Health Organization and UNICEF, 2016).

South Africa is one of the countries that initially opted to provide free formula milk to women living with HIV who chose not to breastfeed. This policy was in place from 2001 until 2011 when, following a national declaration of support for breastfeeding, the provision of free formula milk in government health facilities was phased out (South African Department of Health, 2011). South Africa has historically had very low rates of EBF, even prior to the HIV epidemic. The EBF rate remained at approximately 8% between 1998 and 2013 (Department of Health South Africa and Measure DHS, 2004). The latest demographic and health survey in 2016 (National Department of Health/Statistics South Africa/South African Medical Research Council and ICF, 2019) measured 32% EBF up to the age of 6 months, plausibly due to the policy change in support of breastfeeding. However, considerable improvement is required to reach the global nutrition target (WHO/UNICEF, 2014).

Restoring confidence among health workers to support breastfeeding is especially important in countries with a high HIV prevalence. This remains a considerable challenge; research from several settings has documented confusion among health workers on how to advise and support women to breastfeed, due to incomplete training coverage and weak supervision (Chinkonde, Sundby, de Paoli, & Thorsen, 2010; Chopra, Doherty, Jackson, & Ashworth, 2005; Janse van Rensburg, Nel, & Walsh, 2016; Piwoz et al., 2006). In addition, attitudes and fear among health workers have also contributed to inappropriate infant feeding messages and reluctance to promote breastfeeding, particularly sustained breastfeeding, among mothers living with HIV mothers due to the role breastfeeding has in post‐natal HIV transmission (West et al., 2019).

The availability and high coverage of antiretroviral therapy (>95%; UNICEF, 2016) for pregnant and breastfeeding women living with HIV, together with a supportive infant and young child feeding policy in South Africa, provides a suitable context for supporting breastfeeding. Nonetheless, challenges remain in restoring confidence among health workers to recommend breastfeeding and to support women experiencing common infant feeding difficulties (Ijumba et al., 2013; van Rensburg et al., 2016). This qualitative paper presents findings from focus group discussions (FGDs) with mothers of young infants, to explore their experiences of antenatal and post‐natal infant feeding counselling and support received in PHC settings, including from health facilities and community providers. This paper presents mothers' perceptions of the information and support they received and their experiences of health worker responses to breastfeeding difficulties and challenges in the early post‐natal period.

## 2 METHODS

### 2.1 Study design

This qualitative study was part of a larger quasi‐experimental feasibility study of a mentorship intervention to improve confidence and knowledge of health workers to deliver infant feeding counselling. Health workers involved in infant feeding counselling in 23 intervention clinics across two South African districts (Tshwane and Ugu, respectively, situated within Gauteng and KwaZulu‐Natal Provinces) received three on‐site participatory workshops addressing knowledge gaps and an explanation of the updated infant feeding policy. The three sessions were held weekly and lasted about 1 hr.

Approximately 3 months after the intervention, 12 of the intervention clinics (six in Tshwane and six in Ugu) were purposively selected to participate in FGDs to explore mothers' experiences of infant feeding counselling. The selection of clinics was determined by the number of mothers attending with infants under 6 months of age and availability of physical space to hold a FGD. Although all included facilities had participated in the mentorship intervention, some mothers participating in the discussions may not have received counselling from a health worker who participated in the intervention due to the timing of individual women's antenatal and post‐natal care in relation to the delivery of the intervention. The focus of this qualitative study was not to assess the intervention but to gather infant feeding counselling experiences of mothers with young infants in order to inform recommendations for refining the intervention content and methods of delivery.

### 2.2 Study sites

Two districts, Tshwane (urban, in Gauteng Province) and Ugu (rural, in KwaZulu‐Natal Province), were selected for their differing historical infant feeding contexts: KwaZulu‐Natal has a history of strong political will to support breastfeeding (Horwood et al., 2018), whereas in Gauteng, women living with HIV historically practised predominantly formula feeding until the national declaration of support for breastfeeding in 2011. In PHC clinics, women receive infant feeding information antenatally mainly from nurses and lay counsellors and sometimes nutritionists. Postpartum in health facilities, nurses provide support for breastfeeding initiation, and at clinic level, post‐natal monitoring of infant feeding is included in routine post‐natal care as part of growth monitoring and well child services. In both districts, household‐level information and support can also be received from community health workers (CHWs) who are part of ward‐based outreach teams who visit and screen households for health conditions requiring referral. According to the national guidelines, each ward‐based outreach team consisting of six to eight CHWs should cover an average population of 6,000 individuals or approximately 1,500 households per annum. There are some differences in these ratios across provinces. Standardised CHW training covers identification of the need for antenatal and post‐natal care, monitoring immunisation of under 5 s, adherence among patients with chronic diseases, screening for malnutrition, tuberculosis, gender‐based violence, and making referrals to health, social, and other services (South African National Department of Health, 2015). A focus of the household visits is on pregnant women and children under 5 years where infant feeding counselling, screening for signs of illness, and review of the Road to Health booklet are key tasks.

### 2.3 Participants and data collection

Within the selected clinics, six to eight mothers, regardless of their HIV status, with an infant aged less than 6 months, were approached to participate in a FGD. A FGD guide, with key open‐ended questions and probes, was developed and translated into isiZulu and Tswana. Each group was led by two experienced South African qualitative researchers fluent in the local languages of isiZulu and Tswana. We asked mothers about their experiences with infant feeding, facility and community level support received (both antenatal and post‐natal), and their understanding of information conveyed. The FGDs were undertaken first in Ugu and then in Tshwane to ensure that the reflections and emerging discussion themes from Ugu could be explored in the second district. Following initial analysis of the 12 FGDs, it was determined that further data collection would be unlikely to yield meaningfully different findings as commonly recurring themes had been identified and could be applied to the working analysis framework.

### 2.4 Data analysis

**Figure 1:**
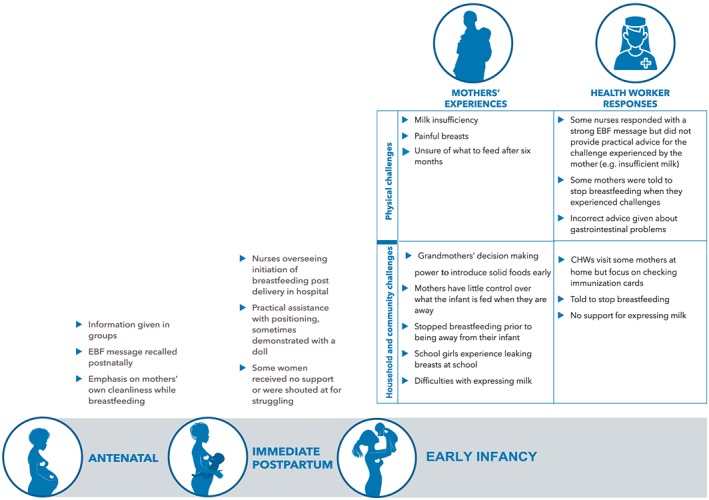
Framework of study findings. CHW, community health worker; EBF, exclusive breastfeeding

Audio‐recorded FGDs were translated into English and transcribed. Transcripts were validated against the recording to ensure that accurate meaning had been captured during translation. Transcripts were imported into NVivo software (QSR International, https://www.qsrinternational.com/nvivo/home) for data management and analysis was based on the framework approach (Gale, Heath, Cameron, Rashid, & Redwood, 2013). This approach is a systematic and flexible approach to analysing qualitative data, which is often used in mixed‐method studies and multidisciplinary applied health research. Six researchers from the research team reviewed the transcripts and coded them independently and then came together in a 4‐day workshop to review the codes and agree on a working analytical framework. The framework was then applied to the organisation and interpretation of the data. Logically, the data could be divided into three key time periods (antenatal, immediate postpartum period in health facilities, and early infancy). Within these three time period, common themes emerged around types of challenges experienced by mothers, notably, physical, household, and community challenges. Results are reported in narrative form and presented according to the developed framework (Figure 1).

### 2.5 Ethical considerations

Ethics approval for the quantitative feasibility study and this qualitative component was granted by the Human Research Ethics Committee of the South African Medical Research Council, the University of KwaZulu‐Natal Biomedical Research Ethics Committee (BREC), and the local department of health research committees in Gauteng and KwaZulu‐Natal. Project staff individually affirmed that they would abide by data security and confidentiality principles and procedures. All mothers approached to participate in an FGD were read an information sheet outlining the expectations in terms of length of the discussion, the voluntary nature of the participation, and measures to ensure confidentiality. Mothers who agreed to participate signed an informed consent form.

## 3 RESULTS

### 3.1 Description of participants

A total of 67 mothers participated in the FGDs with an average of six women per group. The mean age of mothers was 26 years (range 17 to 42), and the average age of the last‐born infants of these participants was 12 weeks (range 1–28 weeks) at the time of the discussion.

### 3.2 Antenatal period

Mothers described receiving infant feeding information during group education sessions at antenatal clinics. No mothers reported receiving individual counselling on infant feeding. EBF for 6 months was a key message that was recalled by all mothers (Box 1). Some mothers reported being given more practical and biological information, whereas others recalled only the EBF key message. Mothers living with HIV recalled specific messages related to the importance of EBF to reduce the risk of transmission. Women were also given considerable information about cleanliness and hygiene in relation to breastfeeding. This included handwashing and washing of breasts and body of the mother. No mothers mentioned receiving information about coping with common breastfeeding challenges such as engorgement, insufficient milk, or painful breasts. Many mothers found the information received during antenatal care helpful, especially first‐time mothers, and they saw nurses as an important source of trustworthy information on infant feeding.

Box 1 **Mothers' perceptions of antenatal infant feeding information**

**Format for communication**

“They advised us in groups when we came for antenatal clinic. They taught us about breastfeeding the baby and how it helps to protect the baby. What are the benefits of breastfeeding and how does the baby gets protected.” (District A, Clinic 4)

“Eh when they advised us we were … it was a class, there were many of us. The sister came and explain to all of us. She even showed us how to breastfeed the baby, how to hold the baby.” (District A, Clinic 5)

**EBF message**

“I was told that the baby must be breastfed until 6 months. You must feed him breastmilk only and do not mix it with anything.” (District A, Clinic 2)

“…. The information that we receive here assists us to know that we should not give babies porridge because breastfeeding alone is fine. She breastfeeds until she is full. Porridge is not recommended until the baby is at least six months old.” (District B, Clinic 3)

“I breastfeed him because the nurses at the clinic told us that it is better to breastfeed the baby because he will not get disease.” (District A, Clinic 4)

**Breastfeeding and personal cleanliness**

“It is important to clean your breast all the time while you still pregnant, clean all dirt, clean it all the time so that the milk can come out. The nurse told me that it is important to clean it … you must clean them while pregnant, clean them always, there is always dirt in them.” (District A, Clinic 6)

**Exclusive breastfeeding and HIV**

“They said if you are breastfeeding you are not supposed to feed the baby other food except breastmilk until he's 6 months. They said since we who are positive, it easy for him [the baby] to be infected with the virus.” (District A, Clinic 5)

### 3.3 The immediate postpartum period in health facilities

Mothers reported receiving varying levels of support following delivery (Box 2). Some mothers received information about breastfeeding practices and demonstrations on positioning including the use of demonstration dolls as aides. A few women reported negative experiences with health workers, such as being shouted at because they were struggling with initiating breastfeeding.

Box 2 **Mothers' experiences postpartum**

**Help, counselling, and demonstrations at the hospital**

“They did watch me because I gave birth through C‐Section. If you give birth through C‐section, you lay on your back then you breastfeed your baby. They do check you if you are breastfeeding correctly or incorrectly.” (District A, Clinic 2)

“Yes, there was a sister who watched me at the hospital while I was breastfeeding, I wasn't discharged. I was breastfeeding incorrectly. Then she corrected me on how should I hold so that he can feed well.” (District A, Clinic 5)

“Yes, after I gave birth the nurse came and sat with me, she watched me and told me that I must hold my breast with four fingers under the breast, one on top so that the baby will be able to get milk properly.” (District A, Clinic 4)

**No help or negative experiences**

“On the first day it was a bit difficult as it was my first born, one doesn't know how to breastfeed nor to hold the baby. It was difficult in a sense that you don't even know for how long the baby should be breastfed. There was no one who could explain to me. What they do is just to hand over the baby and ask you to breastfeed. After that they leave. I asked them how. They just put the baby on my breast and the baby was so tiny, I was unable to hold him. I did not know what to do.” (District B, Clinic 3)

“She yelled at me, she even came to me and pulled my nipple telling me that I'm failing to breastfeed the baby. She told me to put my breast in baby's mouth. I would put it. I would say, there is nothing coming out. She said, there is no such thing. She would hold it like this (demonstrating), squeeze it, milk will come out then she told me to put my breast in the baby's mouth, it couldn't go in then she would take that little container that we used to express milk for the babies, she expressed in that container and give him a little drop. She then stopped and said night shift nurses will check on him.” (District A, Clinic 4)

Other women reported receiving less help and support after delivery—either just a quick message about breastfeeding or no message at all, no help or demonstrations, and then being left to themselves.

### 3.4 Early infancy

#### 3.4.1 Physical challenges with breastfeeding and health worker responses

Mothers described major challenges with breastfeeding after discharge from hospital, including painful breasts and perceived milk insufficiency, and many mothers described inadequate health worker responses (Box 3).

Box 3 **Mothers' descriptions of physical breastfeeding challenges and health worker responses**

Perceived milk insufficiency

“Initially it was difficult because there was little milk coming out, so when I arrived at home I would prepare a bottle for him because whenever I was breastfeeding my breasts would be sore. So, I was unable to breastfeed. So, I bought milk for him. He drinks from the bottle twice a day” (district B, clinic 1)

“Since his birth I've been trying to breastfeed thinking that milk will be there but no there is nothing. I tried to formula feed him because I would breastfeed him for a long time and when I take him out of my breast to feed him bottle he would feed the same way as if he still on breast, which means that milk coming from here (breasts) is too small.” (District A, clinic 4)

Responses to perceived milk insufficiency

“They never helped me. They called me isigqala. I'm like that cow called isigqala which means I do not have milk. It is common for a person to not have milk, they said I must stop him” (district A, clinic 4)

“Whenever I express it takes long to have enough and when he's hungry he would breastfeed, then stop and cry. The nurses advised me to stop breastfeeding because my breast have little milk, he would end up getting sick.” (district A, clinic 4)

“It sometimes happen that the nurse does not have time for you. No, even if you are inside the consultation room and explain your problem, she does not attend you the right way and sometimes she do not give you anything at all” (district A, clinic 2)

“When she finished 5 months, I fed her because I noticed that she's not getting full and her weight had dropped. I did come (to the clinic) they said I should not give her other foods. I said but I can see her, she does not get full from breastmilk only. I decided on my own that I must cook porridge for her.” (district A, clinic 5)

Painful breasts

“I had a problem, my milk was not coming out properly, and my nipples were painful, it would be like they are cracking.” (district A, clinic 6)

“I stopped him when he was still very young, he was one week because I had sores all over my breasts and they were very painful” (district A, clinic 6)

Response to painful breasts

“I told them at the clinic (about painful breasts) and they said I must continue with breastfeeding, when I feel pain and there is blood coming out then I can stop breastfeeding him and give him bottle [formula]” (district A, clinic 6)

Managing alone, hiding the truth

“No I did not come to the clinic because they told us not to feed the baby (solid foods) so I secretly fed him (porridge)” (district A, clinic 2)

“I feed the baby, I give him water. At X clinic they asked me what I gave the baby. I gave him water and pap (maize flour porridge)! You cannot tell them the truth. You cannot tell them, but you know you gave him pap” (district B, clinic 4)

Uncertainty on how to introduce complementary feeds around six months

“Yes I am going to ask her. It is the main reason to come to the clinic today because since she is now turning 6 months what am I supposed to do since they said you must breastfeed for 6 months if you are [HIV] positive. Then I want to know what must I do if I do not have money to buy milk (formula milk)” (district A, clinic 5)

Some mothers described approaching health facilities for help where their difficulties were met with varying responses from health workers. Whereas some nurses continued to advocate for and support EBF, others validated mothers' perceptions of not having enough milk and advised them to stop breastfeeding. Mothers' described that for any reported stomach problems in their infants, health workers advised them to change to formula milk or use glucose water.

Advice from health workers commonly resulted in the introduction of other feeds and some women stopping breastfeeding. Many mothers described giving water and formula immediately after being discharged from the hospital and introducing solid feeds early.

However, some mothers reported managing many of their difficulties on their own without looking for support. As a result of the strong EBF messages received at health facilities, mothers reported hiding formula and were fearful to tell nurses if they had introduced other foods prior to 6 months.

Mothers who practised EBF were unsure how to introduce complementary foods when their infants neared 6 months because of fears surrounding the negative perception of mixed feeding (continuing breastfeeding while adding complementary foods). That was particularly the case for mothers living with HIV who strongly recalled the messages about the importance of not mix feeding to reduce the risk of HIV transmission.

### 3.5 Household and community challenges to infant feeding and health system responses

The focus groups revealed that many mothers struggled to carry out their intended feeding practices due to pressure from family members, especially the infants' grandmothers, to introduce other fluids and foods. Although CHWs were present in the districts where this study took place, few mothers reported being visited at home by a CHW, and those who did receive a visit reported that it was mostly focused on checking whether immunizations were up to date in the Road‐to‐Health booklet. A few mothers, however, did receive support for breastfeeding in their homes and found this to be very helpful.

Mothers reported that they usually did not have the decision‐making power to prevent their infants being introduced to other foods, particularly during periods of absence from their babies. Grandmothers also regarded themselves as people who know best because they have raised children before and are often the primary caregivers for infants whose mothers have returned to work or school. There were a few cases where mothers reported that their mothers were supportive of breastfeeding and gave helpful support and advice (Box 4).

Box 4 **Post‐natal challenges and community‐based health system support**

Post‐natal feeding challenges in the home setting

Power of infants' grandmothers

“My mother is the one who came up with an idea of the porridge. She said milk is just water not food” (district B, clinic 5)

“But the grandmothers also make us dumb, they confuse us because they are aware we go to work or school, so when we get back the baby is not crying, when you ask, they tell you I gave him milk. When you ask those that were at home they will tell you grandmother cooks him porridge. When you ask her “mum why do you give a baby porridge?” she gets upset with you.. Our babies are destroyed by their grandmothers that we leave them with” (district B, clinic 4)

“Grandmothers disagree with the advice that we receive from the hospital. To tell you the truth, they ask us how we grew up but you cannot argue with her because those were the old ways of doing things and there were not as many diseases then as there are now. You find that she will tell you to give the child porridge. We fight every day. I tell her no, and tell her that at the hospital I was told that the child must not eat anything else until she is six months old. It is just that they try to force us to use the old ways that they do not work anymore” (district B, clinic 3)

Positive experiences of community‐based health system support for feeding

**“**Being visited by a CHW at home is very helpful because I never got advice when I was pregnant but getting a visit from a CHW helped me” (District A, clinic1)

“It is helpful, she tells me what must I do and not do for the baby and how to breastfeed the baby. Like I should not change the baby from breastfeeding to formula feeding and that there is three kinds of milk within the breastmilk. The first one quench thirst, the second one cleanse the interior, the third one makes him [baby] full. So if you change the baby from breast he will not get full.” (district A, clinic 6)

“When maybe I forget how to hold my breast she corrects me on how to hold the breast correctly so that the baby can feed nicely. She's my neighbour” (district A, clinic 2)

Community‐based visits without infant feeding support

“I think it was right because she (the CHW) would come in my home, open my baby's card, check if all the immunization for the baby are in order and when is my next visit date to the clinic. She has never told me anything about breastfeeding” (district A, clinic 4)

“She first check the card, check the date for my next clinic visit, then sign and then go to another household” (district A, clinic 2)

Another challenge that many mothers described in the post‐natal period was returning to work or school. Mothers who had returned to work or school described wanting advice and support from nurses about maintaining breastfeeding during periods of absence. Some mothers returning to work or school said they were advised by nurses to switch to formula milk. The common challenges of expressing milk and maintaining breastfeeding during periods of absence did not appear to be responded to during clinic visits, and mothers opted to take the advice of those looking after their infants or stopping breastfeeding in advance of returning to work (Box 5).

Box 5 **Challenges experienced with returning to school or work and health worker responses**

**Challenges experienced by mothers when returning to school or work**

“He is younger than one month. I give him formula. She (grandmother) is the one who takes care of the baby when I am at work. In the evenings I breastfeed him. During the day he drinks formula” (district B, clinic 4)

“I now go to work and come back in the afternoon (infant aged 3 months). I start working at 7 and I knock off at 4. I express the milk for him to feed during the day but he does not want it, it stays all day long until I come back. He waits for me until I come back. My mother was feeding him porridge but you can see that he only ate small amount, he did not want to feed on the bottle” (district A, clinic 2)

“I think I should start feeding my baby because I want to go back to school soon. I will express but I know it will not be enough because I breastfeed him often. So when I leave him at home with the person taking care of him she should feed him.” (district B, clinic 2)

“The only challenge I have is that my breasts get full and start leaking when I am at school. The breasts get so full that I have to express the milk from them.” (district B, clinic 5)

“He eats. He is two months old. My mother told me that I must prepare a bottle for him. When I go to school I express breast milk for him. My mother said the baby will get sick easily if he only drinks formula. That is why I express the milk for the baby when I go to school. I give him porridge and breast milk.” (district B, clinic 5)

“I struggle to express milk. I heard that I have to express the milk into a bottle. I am a cook. I am about to return to work (infant aged 4 months). I lose patience because very little milk comes out.” (district B, clinic 5)

Mothers' perceptions of health worker responses to coping with feeding during periods of absence

“I wanted to stop breastfeeding and had asked at the clinic and mentioned that I found a job. They said I can give the baby formula” (district B, clinic 2)

“The problem is that at the clinic they do say you can give bottle [formula milk] to the baby. When you are going somewhere you are able to leave him with bottle [formula]” (district A, clinic 6)

“It was written on my antenatal card that I will give my baby formula milk because I will not stay with him. When I asked for formula milk after I gave birth they changed and said the baby must have breastmilk, I then breastfed the baby. They wrote without asking me but after they have written they told me that breastmilk is very important to the baby. I also thought that if I continue to breastfeed my baby he will not be able to formula feed when I stop breastfeeding him. I stopped him, he only breastfed when I was in hospital for 3 days then I stopped when I got home.” (district A, clinic 3)

The most preferred option described by women returning to work was to stop breastfeeding and switch to formula feeding prior to returning to work. Few women described expressing breastmilk to leave for their infants while at work or school. Most of the mother's describing returning to work or school were from the urban Tshwane district. Their concerns were related to not being able to express sufficient milk for the day and fears that family members would give other foods in addition to the expressed breastmilk and they reported having very little control over what their infants were fed while they were away.

Young mothers who were returning to school experienced difficulties with breasts leaking while at school, which required expressing milk during school to relieve breast engorgement (Box 5).

## 4 DISCUSSION

This qualitative study provides insights into mothers' experiences of support received for infant feeding in PHC settings after policies had shifted to supporting breastfeeding in the context of more effective interventions to prevent mother to child transmission of HIV. The findings highlight that mothers do receive group information during antenatal care with a major focus on the importance of 6 months of EBF, but these sessions did not prepare mothers for the challenges they were likely to face in maintaining EBF. Post‐natally, mothers described receiving some practical support with initiation of breastfeeding after delivery, but support for common challenges in the early post‐natal period appeared to range from supportive and correct messages to incorrect or absent advice by health workers. The early post‐natal period is a critical time to establish and support breastfeeding, and these findings underscore the need for continued, home‐based and clinic‐based support for mothers when they experience common challenges.

Although health workers did emphasize EBF through a clear and consistent message, our findings suggest that the interactions appeared to be largely limited to this message without proactive guidance on other aspects of breastfeeding, especially how to deal with challenges and periods of transition. An approach could be for health workers to provide mothers with specific information and support to develop their self‐efficacy in overcoming likely challenges, for example, messages to counter contrary advice from family members. In addition, the focus on exclusivity meant that women, especially mothers living with HIV, were unsure about how to introduce complementary foods around 6 months due to fears of mixing breastmilk with solid food. It could even result in advising a mother to switch to formula milk if she was not exclusively breastfeeding. Such echoes from previous national guidelines need to be managed in the retraining of health care providers to avoid a detrimental impact on child health. A recent qualitative study undertaken in Johannesburg (West et al., 2019) also found that mothers living with HIV expressed confusion around continued breastfeeding after the introduction of complementary foods. This confusion was pervasive among both mothers and health care providers. Counselling given in the antenatal clinic (ANC) was usually in a group setting making it difficult for mothers' individual concerns to be addressed, particularly for mothers living with HIV. This underscores the need for ongoing supervision and refresher inputs, including reinforcement of knowledge and clinical skills needed to assist women with common challenges and periods of transition. Furthermore, all mothers should have an opportunity to receive individual counselling during ANC.

Recently published findings from the “Alive and Thrive” intervention in Burkina Faso (Cresswell et al., 2019; Rollins & Doherty, 2019) show how a multidimensional intervention, including training and supervision of facility‐based and community‐based health workers, together with community mobilization activities, led to substantial increases in EBF and improvements in the knowledge and beliefs of mothers concerning breastfeeding. This study adds to the growing evidence base concerning the need for breastfeeding interventions to be multifaceted including facility, community, household, and workplace dimensions (Rollins et al., 2016). Although in South Africa, there is an attempt to complement health facility‐based care for families through deployment of CHWs, our study has found that although some participants received post‐natal home visits from CHWs, they may be focused more on the task of checking immunization coverage than assessing household needs and providing support. Previous research in South Africa has shown that home‐based peer breastfeeding counselling can have a significant effect on improving breastfeeding practices (Ijumba et al., 2015; Tomlinson et al., 2014; Tylleskar et al., 2011). Home‐based support also offers the opportunity to bring other family members and key decision makers into discussions, which is vital because this and other research have shown the powerful role played by grandmothers in infant feeding practices (Ijumba et al., 2014). An effective continuum of care from facility to community for women with young infants will require a far higher ratio of CHWs to population served, as well as enhanced training and supervision (Doherty, Kroon, Rhoda, & Sanders, 2016).

Our research also found that the support provided by health workers appeared blind to the context in which women live and make decisions around infant feeding. Women, in the South African context, who are often single and living separately from their partner (National Department of Health/Statistics South Africa/South African Medical Research Council and ICF, 2019), face pressures from family members who have a strong influence on how they feed their children, and the counselling from health workers appears not to prepare them for these challenges or, even in some instances, resorts to victim blaming of mothers for not following their advice. The mothers' experiences in our study also highlight the power imbalance and social distance between clients and health workers. This was evidenced from mothers' accounts of hiding their feeding methods (when not exclusively breastfeeding) from health workers for fear of reproach. A similar finding was reported by a qualitative longitudinal study among adolescent mothers in two different districts of KwaZulu‐Natal, where participants reported not revealing how they were truly feeding their infants to avoid conflict with health workers (Jama et al., 2018). These situations can also lead to incorrect recording of feeding practices in routine health information, thus underestimating the need for health worker training and supervision.

The training curriculum and materials related to infant feeding for both nurses and CHWs require strengthening particularly with regard to skills for dealing with breastfeeding challenges. Further, we believe these experiences bring to light the many pressures mothers experience from families, health systems, and work places and urge governments to operationalize policies focusing on breastfeeding in work places and women's wider rights for job security that does not undermine breastfeeding.

### 4.1 Strengths and limitations

Our study included 12 FGDs with mothers of young infants across two districts and therefore gives a rich picture of mothers' experiences of infant feeding counselling at PHC facilities. The groups were facilitated by experienced qualitative researchers in the home language of participants. The clinics where the FGDs were held were selected to obtain sufficient numbers of mothers with young infants. There was no specific attempt to try and recruit mothers who had received counselling from nurses who participated in the mentorship intervention. We hypothesized that the intervention would not have direct impact on these mothers' feeding practices because of the timing of the FGDs being only 3 months after the intervention, when many of these infants would already have been born. Thus, we focused on understanding feeding practices in the general population of mothers and infants in order to further inform and refine our recommendations for future mentorship interventions.

We did not interview health workers in order to understand their experiences of counselling women; we only present mothers' experiences of the care they received from health workers. In the South African PHC system, health workers are commonly overburdened with large numbers of clients presenting with multimorbidities, and low morale and motivation is a pervasive challenge. Achieving high quality of care across the spectrum of tasks at PHC level within such contexts requires skilled leadership and ongoing supportive supervision.

## 5 CONCLUSIONS

Despite improved breastfeeding policies, restoring confidence in health workers to support breastfeeding remains a challenge. The post‐natal period, when mothers experience practical breastfeeding difficulties, is particularly critical, and our findings reinforce the importance of continuity of care between communities and health facilities. This research has implications for how facility and community‐based health workers are trained and supervised to support breastfeeding. Greater attention is needed on developing health worker skills and confidence in identifying, assessing, and supporting women experiencing breastfeeding challenges.

## CONFLICTS OF INTEREST

The authors declare that they have no conflicts of interest.

## CONTRIBUTIONS

TD, CH, AG, LH, and IE participated in the conceptualisation of the study. VM participated in the collection of the data. TD, AG, LH, CH, UF, DS, and IE participated in the analysis and interpretation as well as writing. All authors were involved in reviewing the manuscript and approved the final version of the paper.
